# Health and Safety Executive Management Standards: associations with operational effectiveness in policing

**DOI:** 10.1093/occmed/kqaf018

**Published:** 2025-03-19

**Authors:** Jonathan Houdmont

**Affiliations:** School of Medicine, University of Nottingham, Nottingham NG8 1BB, UK

## Abstract

**Background:**

The United Kingdom (UK) Health and Safety Executive’s Management Standards encompass a set of aspirational quality standards and a risk management methodology pertaining to psychosocial working conditions. Two decades since their introduction, implementation of the Management Standards or equivalent approaches remains far from universal across UK organizations. This may be due, in part, to a paucity of evidence concerning their operational effectiveness benefits.

**Aims:**

This study aimed to generate evidence on the business benefits of the Management Standards by examining associations between achievement of the good practice quality standards and indices of operational effectiveness.

**Methods:**

Police custody sergeants (*N* = 1493) completed the Management Standards Indicator Tool that assesses the extent to which the quality standards are met, plus measures of operational effectiveness (job performance, attendance behaviours, intention to leave). Logistic regression was used to examine associations between achievement of the quality standards and operational effectiveness.

**Results:**

The proportion of respondents reporting fulfilment of the quality standards in their workplace ranged from 3% (change) to 65% (role). Achievement of the quality standards was variously associated with elevated odds for the concurrent presence of desirable states of operational effectiveness.

**Conclusions:**

These findings point to the operational effectiveness benefits of a preventative approach to the management of workplace psychosocial risk and may encourage organizations to adopt the Management Standards or an equivalent approach to fulfil their legal duty in respect to psychosocial risk management.

## Teaser

The UK Management Standards support organizations in the fulfilment of their legal duty to manage psychosocial risk, yet adoption remains far from universal. This may be due to lack of awareness of operational effectiveness benefits. This study provides evidence on associations between the Management Standards’ psychosocial work environment quality standards and indices of operational effectiveness that hold implications for the ‘bottom line’. Findings may help practitioners make business-based arguments to embed preventative psychosocial risk management.

**Key learning points**

**What is already known about this subject**

- The UK Health and Safety Executive produced its Management Standards to help organizations fulfil their duty to manage psychosocial risk; these encompass a set of aspirational quality standards and a risk management methodology pertaining to psychosocial working conditions.
- Two decades since their introduction, implementation of the Management Standards or equivalent procedures remains far from universal across UK organizations; this may be due, in part, to a paucity of evidence concerning their operational effectiveness benefits.

**What this study adds**

- This study builds on the existing evidence base by generating data on the business benefits of the Management Standards by examining associations between achievement of the aspirational quality standards and six indices of operational effectiveness in a large sample of police custody sergeants.
- Achievement of the Management Standards’ aspirational quality standards was variously associated with elevated odds for the concurrent presence of desirable indices of operational effectiveness.

**What impact this may have on practice or policy**

- These findings highlight the operational effectiveness (‘bottom line’) benefits of a preventative approach to the management of workplace psychosocial risk and may encourage organizations to adopt the Management Standards or an equivalent approach to fulfil their legal duty in respect to the management of psychosocial risk.

## Introduction

The United Kingdom (UK) Health and Safety Executive (HSE) produced the Management Standards to support employers in the fulfilment of their legal duty to take preventative steps to manage psychosocial risk [1, 2]. This approach comprises a psychosocial risk management procedure centred on key areas of work design—demands, control, support, role, relationships, and change—with an aspirational good practice quality standard defined for each. To help organizations assess the extent to which each quality standard is achieved and establish whether further control measures are required to reduce risk of harm, the HSE recommends its self-report survey instrument, the Management Standards Indicator Tool (MSIT) [3].

UK and European campaigns have sought to raise employer awareness of the duty to manage psychosocial risk and the availability of psychosocial risk management methodologies such as the Management Standards. Yet knowledge and implementation of psychosocial risk management remains far from universal. A 2023 survey of 438 UK organizations found that 20% used the Management Standards, while 58% reported using ‘risk assessments/stress audits’ [4]. The 2019 ESENER-III survey revealed that across 2251 participating UK organizations fewer than 60% felt they had sufficient information on how to include psychosocial factors in risk assessments and around one third did not have a work-related stress action plan [5]. Meanwhile, a 2014 survey of 804 UK occupational safety and health practitioners found that amongst those with responsibility for psychosocial risk management (around 50% of the sample), almost half reported having not yet undertaken any such activity [6].

Knowledge of factors that drive organizational health and safety (OSH) activity can provide guidance on the type of evidence that might leverage the adoption of psychosocial risk management approaches such as the Management Standards. Awareness of operational effectiveness gains—or business benefits—is a key driver of OSH activity, yet findings from ESENER-III led the authors to conclude that ‘the business case or value of OSH on the bottom line is not fully appreciated or understood by industry’ (p.93) [5]. This was evident, for instance, in the identification of ‘increasing productivity’ as the least important factor among a list of five drivers of OSH activity. Consistent with this, only 33% of 438 UK organizations surveyed in 2023 identified ‘improve performance’ as an opportunity arising from investment in employee health and well-being [4].

Evidence for operational effectiveness benefits linked to fulfilment of the psychosocial work environment good practice quality standards set out in the Management Standards may support their adoption. There is a paucity of such evidence, with most studies having examined linkages between MSIT scores and indices of employee mental health such as burnout [7–9], mental well-being [10–12], and psychological distress [13–15]. While these studies demonstrate the relevance of the Management Standards to workforce health and well-being, less attention has been paid to linkages with aspects of operational effectiveness that may possess more overt implications for business operations and the ‘bottom line’. Within this literature, a limited number of studies have examined associations with indices of operational effectiveness such as job performance, attendance behaviours, and intention to leave [16–20]. Taken together, these studies have demonstrated mostly small or negligible linkages between MSIT scores and operational effectiveness, potentially undermining likely assumed business benefits associated with a high quality psychosocial work environment.

Accordingly, the aim of the current study is to expand the research base on potential business benefits arising from the Management Standards. This is achieved by examining associations between fulfilment of each of the Management Standards’ aspirational psychosocial work environment quality standards and six indices of operational effectiveness: specifically, three forms of job performance, two attendance behaviours, and intention to leave the occupation in a sample of police officers.

## Methods

Data reported here are drawn from a study concerning the work and health of police custody sergeants in England and Wales that was conducted during 2013 and 2014 [21, 22]. Police custody sergeants manage a custody suite and hold responsibility for the care and welfare of detained persons and the decision to authorize or refuse the detention of persons presented before them. Police Federation representatives informed eligible officers about the study via an email containing an invitation to participate and a hyperlink to an online survey. Participation was voluntary and anonymous, with ethical approval granted by the research ethics committee of the Faculty of Medicine and Health Sciences, University of Nottingham.

The extent to which the Management Standards’ psychosocial work environment aspirational quality standards were met was assessed using the 25-item version of the Management Standards Indicator Tool (MSIT-25) [23]. The MSIT-25 is commonly used where an imperative exists for brevity in survey length [19, 24] and its acceptability as an alternative to the original MSIT-35 is psychometrically established [23, 25–27]. Responses to the first 15 items are given on a five-point scale ranging from never (1) to always (5), with negatively framed items (e.g. ‘I have unachievable deadlines’) reverse scored so that low scores indicate poor psychosocial working conditions. Responses to the remaining items are given on a five-point scale of strongly disagree (1) to strongly agree (5), with negatively framed items (e.g. ‘I am subject to bullying at work’) reverse scored. A mean score was generated for each Standard: demands (4 items), control (4 items) managerial support (5 items), peer support (4 items), relationships (2 items), role (3 items), and change (3 items), with higher scores indicating a better psychosocial work environment. The HSE does not specify a cut-off score for achievement of a quality standard. In the current study, this was defined as score of ≥4, corresponding with a desirable psychosocial work characteristic being ‘often’ or ‘always’ present and an undesirable state being present ‘never’ or ‘rarely’.

Job performance concerns ‘things that people actually do, actions they take, that contribute to the organization’s goals’ (p. 48) [28]. This implies three notions: performance concerns behaviours rather than results, encompasses behaviours that contribute to the organization’s goals, and is multidimensional [29]. These notions point to a distinction between task performance and contextual performance, with the former referring to core in-role behaviours concerned with fulfilment of specified requirements of the job, while the latter concerns citizenship behaviours directed at individuals and the organization. These discretionary extra-role behaviours play an important function in creating a co-operative social context that supports others’ performance of core responsibilities. This conceptualization of job performance has been widely used in organizational research, including that involving police officers [30], and is assessed herein using a 21-item scale developed by Williams and Anderson [31]. This measures core in-role performance behaviours (IRB: 7 items), e.g. ‘I fulfil responsibilities specified in my job description’; organizational citizenship behaviours targeted at individual colleagues which indirectly benefit the organization (OCBI: 7 items), e.g. ‘I go out of my way to help new colleagues’; and organizational citizenship behaviours that directly benefit the organization (OCBO: 7 items), e.g. ‘I adhere to informal rules devised to maintain order’. Responses were recorded on a seven-point scale ranging from strongly disagree (1) to strongly agree (7), with negatively framed items reverse scored to ensure that higher scores indicate better performance. For each dimension, a sum score was calculated and dichotomized using the median split to identify workers reporting above average performance.

Intention to leave one’s occupation was assessed using a measure originally developed for use in policing [32] that requires respondents to select one response from among six options that indicate varying degrees of intention to leave: ‘I would not change from being a police officer for anything in the world’ (1), ‘I can’t think of any job I would prefer’ (2), ‘I would like to change my posting but remain a police officer’ (3), ‘I would like to change my job and stop being a police officer’ (4), ‘I would take almost any other job with similar earnings and benefits’ (5), and ‘I would quit immediately if I could find something else to do’ (6). Responses of 1–3 were combined to form a ‘no intention to leave’ category.

Two work attendance behaviours were assessed: leaveism and sickness absence. Leaveism is a broad construct encapsulating several work attendance behaviours [33], with most research having focused on the utilization of allocated time off (e.g. annual leave, flexi hours, banked re-rostered rest days, etc.) to take time off when sick. Previous research has revealed a high prevalence of this form of leaveism in English and Welsh policing [34]. To quantify leaveism and sickness absence respondents indicated the number of days in the last six months that they had (i) used annual leave when sick to avoid a recording of sickness absence and (ii) taken sick leave. Responses to each item were dichotomized into no leaveism/sickness absence versus one or more days. Socio- and occupational-demographic characteristics included age, gender, and constabulary.

Descriptive statistics were calculated for each Management Standard including the mean, standard deviation, and proportion of respondents who reported that their workplace achieved the aspirational quality standard. For each operational effectiveness characteristic binary logistic regression with 95% confidence intervals (CIs) was used to estimate odds ratios for the concurrent presence of desirable states of operational effectiveness (i.e. IRB, OCB-I, OCB-O above the median; no absence; no leaveism; no intention to leave) associated with the achievement of each quality standard (i.e. high-quality psychosocial working conditions) relative to failure to meet each standard (i.e. low-quality psychosocial working conditions). In addition to crude odds ratios, adjusted odds ratios were produced that controlled for the influence of each of the other Management Standard areas. Preliminary correlation analyses indicated that socio-demographic characteristics (age, gender) were not meaningfully associated with indices of operational effectiveness (*r* < .1) and as a such these were not controlled for in logistic regression analyses. Statistical significance was defined as *P* < .05 throughout. Analyses were performed in SPSS Version 28.

## Results

A total of 1493 completed usable surveys were returned. The mean age of respondents was 44 (*SD* 6.42) and 82% identified as male. Reliable data on the total number of custody sergeants employed were available for 27 constabularies, across which the response rate was 43%. Descriptive data concerning the Management Standards’ psychosocial work environment quality standards are shown in Table 1. Most respondents (65%) reported achievement of the ‘role’ quality standard in their workplace, while approximately half (49%) reported achievement of the ‘relationships’ quality standard. Fewer than one in 10 respondents reported achievement of the ‘change’ and ‘control’ quality standards.

**Table 1.** Management standards’ psychosocial work environment quality standards

Standard	Standard definition	Mean (*SD*)	Standard achieved (score ≥ 4) *N* (%)
*Demands*	Employees indicate that they are able to cope with the demands of their jobs	3.39 (0.75)	403 (27)
*Control*	Employees indicate that they are able to have a say about the way they do their work	2.74 (0.79)	100 (7)
*Managerial Support*	Employees indicate that they receive adequate information and support from their superiors	2.86 (0.82)	163 (11)
*Peer Support*	Employees indicate that they receive adequate information and support from their colleagues	3.59 (0.67)	570 (38)
*Relationships*	Employees indicate that they are not subject to unacceptable behaviours, e.g. bullying at work	3.78 (0.88)	727 (49)
*Role*	Employees indicate that they understand their roles and responsibilities	4.05 (0.71)	963 (65)
*Change*	Employees indicate that the organization engages them frequently when undergoing an organizational change	2.35 (0.77)	45 (3)

Regarding job performance, achievement of the ‘demands’ (AOR, 1.40; CI, 1.08–1.80) and ‘role’ (AOR, 1.74; CI, 1.38–2.19) quality standards was associated with elevated odds for the concurrent presence of above average in-role behaviour, after controlling for the influence of the other Management Standards areas. In the same way, achievement of the ‘control’ (AOR, 1.55; CI, 1.00–2.41), ‘managerial support’ (AOR, 1.79; CI, 1.24–2.58), and ‘peer support’ (AOR, 1.79; CI, 1.43–2.25) standards was associated with elevated odds of above average organizational citizenship behaviour targeted at individuals, while achievement of the ‘peer support’ (AOR, 1.53; CI, 1.22–1.92) and ‘role’ (AOR, 1.44; CI, 1.14–1.81) standards was associated with elevated odds of above average organizational citizenship behaviour targeted at the organization (Table 2).

**Table 2.** Achievement of the Management Standards’ quality standards, associations with job performance behaviours

Standard	In-role behaviour (above median)		Organizational citizenship behaviour—individual (above median)		Organizational citizenship behaviour—organisation (above median)	
	OR (95% CI)	AOR (95% CI)	OR (95% CI)	AOR (95% CI)	OR (95% CI)	AOR (95% CI)
Demands	**1.72** ^***^ **(1.37–2.17)**	**1.40** ^**^ **(1.08–1.80)**	1.19 (0.95–1.49)	0.87 (0.67–1.13)	**1.39** ^**^ **(1.11–1.75)**	1.05 (0.82–1.36)
Control	**1.85** ^**^ **(1.23–2.80)**	1.36 (0.88–2.08)	**1.94** ^**^ **(1.28–2.94)**	**1.55** ^*^ **(1.00–2.41)**	**1.67** ^*^ **(1.11–2.51)**	1.26 (0.82–1.94)
Managerial Support	**1.52** ^*^ **(1.10–2.10)**	1.09 (0.76–1.56)	**2.36** ^***^ **(1.68–3.30)**	**1.79** ^**^ **(1.24–2.58)**	**1.74** ^***^ **(1.26–2.42)**	1.27 (0.86–1.76)
Peer support	**1.33** ^**^ **(1.08–1.64)**	1.07 (0.85–1.35)	**2.00** ^***^ **(1.62–2.47)**	**1.79** ^***^ **(1.43–2.25)**	**1.77** ^***^ **(1.43–2.18)**	**1.53** ^***^ **(1.22–1.92)**
Relationships	**1.27** ^*^ **(1.03–1.55)**	1.03 (0.83–1.29)	1.08 (0.88–1.33)	0.92 (0.73–1.14)	**1.25** ^*^ **(1.01–1.53)**	1.05 (0.84–1.31)
Role	**1.97** ^***^ **(1.58–2.46)**	**1.74** ^***^ **(1.38–2.19)**	**1.42** ^***^ **(1.15–1.76)**	1.18 (0.94–1.49)	**1.68** ^***^ **(1.35–2.09)**	**1.44** ^**^ **(1.14–1.81)**
Change	1.43 (0.79–2.59)	0.94 (0.50–1.75)	1.81 (0.99–3.32)	1.00 (0.52–1.90)	1.76 (0.97–3.21)	1.08 (0.58–2.03)

Reference category, quality standard not achieved. OR, crude odds ratio. AOR, odds ratio adjusted for all other quality standards. Significant findings in bold.

^*^ *P* < 0.05.

^**^ *P* < 0.01.

^***^ *P* < 0.001.

For attendance behaviours, none of the quality standards displayed an association with absence in crude or adjusted analyses. However, achievement of the ‘demands’ (AOR, 1.89; CI, 1.29–2.79), ‘peer support’ (AOR, 1.51; CI, 1.10–2.07), and ‘relationships’ (AOR, 1.81; CI, 1.35–2.42) quality standards was associated with elevated odds of zero leaveism, after controlling for the influence of the other Management Standards areas. In the same way, achievement of the ‘control’ (AOR, 1.85; CI, 1.13–3.05), ‘managerial support’ (AOR, 2.25; CI, 1.48–3.42), ‘relationships’ (1.34; CI, 1.08–1.67), and ‘role’ (AOR, 1.33; CI, 1.06–1.67) quality standards was associated with elevated odds of zero intention to leave (Table 3).

**Table 3.** Achievement of the Management Standards’ quality standards, associations with attendance behaviours and intention to leave

Standard	Absence (none)		Leaveism (none)		Intention to leave (none)	
	OR (95% CI)	AOR (95% CI)	OR (95% CI)	AOR (95% CI)	OR (95% CI)	AOR (95% CI)
Demands	1.21 (0.95–1.54)	1.17 (0.90–1.52)	**2.44** ^***^ **(1.71–3.49)**	**1.89** ^**^ **(1.29–2.79)**	**1.75** ^***^ **(1.38–2.22)**	1.20 (0.92–1.56)
Control	1.13 (0.74–1.73)	1.05 (0.68–1.64)	1.27 (0.73–2.25)	0.87 (0.48–1.58)	**2.73** ^***^ **(1.70–4.41)**	**1.85** ^*^ **(1.13–3.05)**
Managerial Support	0.98 (0.70–1.38)	0.90 (0.62–1.30)	1.40 (0.88–2.22)	0.85 (0.51–1.41)	**3.26** ^***^ **(2.20–4.83)**	**2.25** ^***^ **(1.48–3.42)**
Peer support	1.04 (0.84–1.29)	0.99 (0.78–1.24)	**1.78** ^***^ **(1.33–2.38)**	**1.51** ^**^ **(1.10–2.07)**	**1.59** ^***^ **(1.29–1.97)**	1.18 (0.94–1.49)
Relationships	1.11 (0.90–1.37)	1.06 (0.85–1.32)	**2.14** ^***^ **(1.63–2.83)**	**1.81** ^***^ **(1.35–2.42)**	**1.66** ^***^ **(1.35–2.04)**	**1.34** ^**^ **(1.08–1.67)**
Role	1.21 (0.97–1.50)	1.18 (0.93–1.48)	**1.46** ^**^ **(1.12–1.91)**	1.14 (0.86–1.52)	**1.72** ^***^ **(1.39–2.13)**	**1.33** ^*^ **(1.06–1.67)**
Change	0.91 (0.49–1.66)	0.84 (0.45–1.58)	1.03 (0.47–2.23)	0.59 (0.26–1.36)	**2.44** ^*^ **(1.22–4.84)**	1.20 (0.58–2.49)

Reference category, quality standard not achieved. OR, crude odds ratio. AOR, odds ratio adjusted for all other quality standards. Significant findings in bold.

^*^ *P* < 0.05.

^**^ *P* < 0.01.

^***^ *P* < 0.001.

## Discussion

In the context of police custody operations, these findings provide insight into relations between a high-quality psychosocial work environment—represented by achievement of the UK HSE’s Management Standards’ aspirational quality standards—and facets of operational effectiveness. In doing so, they extend the limited body of scientific evidence demonstrating the business benefits of the Management Standards [16–20]. Across the Management Standard areas, there was considerable variance in the proportion of respondents reporting achievement of the quality standards in their work environment. At the bottom end of the range, 3% and 7% of respondents reported achievement of the ‘change’ and ‘control’ quality standards, respectively. In contrast, 49% and 65% reported achievement of the ‘relationships’ and ‘role’ quality standards, respectively. In line with previous MSIT research involving police officers [35], these findings suggest that psychosocial work environment quality across the Management Standards areas was not consistent.

Achievement of the Management Standards’ quality standards was variously associated with elevated odds for the concurrent presence of desirable indices of operational effectiveness. Following adjustment, five of the six measured facets of operational effectiveness (the exception being absence) were associated with achievement of two or more quality standards. Notably, the ‘change’ quality standard displayed no association with any index of operational effectiveness.

Some features of this study need to be considered when interpreting its findings. The cross-sectional design precludes definitive conclusions on the existence of causal relations between variables. Response bias may have been present, with individuals experiencing poor quality psychosocial working conditions viewing participation as an opportunity to communicate dissatisfaction and therefore more inclined to participate, leading to their over-representation. Conversely, individuals experiencing undesirable psychosocial working conditions such as excessive demands may be overwhelmed and less able to make time for survey completion, resulting in their under-representation.

This study involved workers in a specific occupational role and context, that of police sergeant responsible for custody suite management. As such, the findings are influenced by, among other things, the highly prescribed nature of the police custody sergeant role that offers limited scope for control over what work is undertaken, how it is done, and the speed at which work is performed, as well as organization-specific attendance management policies. Indeed, the fact that none of the quality standards demonstrated a significant association with sickness absence, while several did so with leaveism, likely reflects an informal convention of requesting use of annual leave or rest days when ill rather than taking sick leave that was commonplace in policing in England and Wales at the time of data collection [34]. While providing an important illustration of linkages between psychosocial work environment quality reflected in fulfilment of the Management Standards’ quality standards and indices of operational effectiveness, care should be applied in the generalization of these findings to other occupational roles and contexts. Further research involving a wide array of occupations and organizations is required to support the development of a strong evidence base concerning the operational effectiveness benefits of psychosocial risk management that may, in turn, act as a driver of such activity.

MSIT scores reported in this study were broadly comparable with that observed in other studies of UK police sergeants [35] and generally poorer than found in benchmark data for the UK general working population [23]. The mean score for six of the seven quality standards (the exception being ‘demands’) was below that found in the general working population and the mean score for three of these areas fell below the 5th percentile. However, since data collection occurred in 2013 and 2014 it should not be assumed that psychosocial work environment quality reported herein reflects current conditions; indeed, it is also possible that the manner in which working conditions are perceived, and relations with facets of operational effectiveness, have changed in the intervening period. It should further be noted that the general working population dataset was published in 2012, since which time considerable developments in the world of work have occurred—particularly following the Covid-19 pandemic—raising the possibility that benchmark data do not necessarily reflect contemporary psychosocial work environment quality.

In this study, achievement of the ‘management support’ quality standard was associated with more than a doubling of odds of a report of zero intention to leave and 79% increased likelihood of above-average performance on the OCBI domain. Alongside this, the ‘peer support’ and ‘role’ quality standards demonstrated an association with no fewer than three indices of operational effectiveness. These findings highlight the value that is to be found in investment in workplace health and wellbeing initiatives that seek to foster line manager and peer-to-peer support, as well as the importance of ensuring that employees are clear about their duties and responsibilities and how these contribute to departmental and organizational goals.

The HSE advises that in respect to psychosocial risk management, ‘decision makers are usually motivated by one of three arguments; the legal, moral or financial. Constructing your case around each of these three arguments may help you convince the powers that be’ (p.1) [36]. This study represents a contribution to the financial argument and may support practitioners in the development of an evidence-based business case for the implementation of preventative psychosocial risk management activity in the organizations they serve.

## Data Availability

Data are available upon reasonable request from jonathan.houdmont@nottingham.ac.uk
